# Sudden Cardiac Death in Athletes in Italy during 2019: Internet-Based Epidemiological Research

**DOI:** 10.3390/medicina57010061

**Published:** 2021-01-12

**Authors:** Fabrizio Sollazzo, Vincenzo Palmieri, Salvatore Francesco Gervasi, Francesco Cuccaro, Gloria Modica, Maria Lucia Narducci, Gemma Pelargonio, Paolo Zeppilli, Massimiliano Bianco

**Affiliations:** 1 Unità Operativa Complessa di Medicina dello Sport e Rieducazione Funzionale, Fondazione Policlinico Universitario Agostino Gemelli IRCCS, Università Cattolica del Sacro Cuore, 00168 Rome, Italy; fabriziosollazzo.md@gmail.com (F.S.); gervasi.salvatore.md@gmail.com (S.F.G.); francesco.cuccaro87@gmail.com (F.C.); glorymodica@gmail.com (G.M.); paolo.zeppilli@unicatt.it (P.Z.); massimiliano.bianco@policlinicogemelli.it (M.B.); 2 Dipartimento di Scienze Cardiovascolari e Toraciche, Fondazione Policlinico Universitario Agostino Gemelli IRCCS, Istituto di Cardiologia, Università Cattolica del Sacro Cuore, 00168 Rome, Italy; marialucia.narducci@policlinicogemelli.it (M.L.N.); gemma.pelargonio@policlinicogemelli.it (G.P.)

**Keywords:** sudden cardiac death, sport-related death, sport, exercise, athletes, pre-participation screening

## Abstract

*Background and objectives:* An Italian nationwide pre-participation screening approach for prevention of sudden cardiac death in athletes (SCD-A) in competitive sportspeople showed promising results but did not achieve international consensus, due to cost-effectiveness and the shortfall of a monitoring plan. From this perspective, we tried to provide an epidemiological update of SCD-A in Italy through a year-long internet-based search. *Materials and Methods:* One year-long Google search was performed using mandatory and non-mandatory keywords. Data were collected according to prevalent SCD-A definition and matched with sport-related figures from Italian National Institute of Statistics (ISTAT) and Italian National Olympic Committee (CONI). *Results:* Ninety-eight cases of SCD-A in 2019 were identified (48.0% competitive, 52.0% non-competitive athletes). Male/female ratio was 13:1. The most common sports were soccer (33.7%), athletics (15.3%) and fitness (13.3%). A conclusive diagnosis was achieved only in 37 cases (33 of cardiac origin), with the leading diagnosis being coronary artery disease in 27 and a notably higher occurrence among master athletes. Combining these findings with ISTAT and CONI data, the SCD-A incidence rate in the whole Italian sport population was found to be 0.47/100,000 persons per year (1.00/100,000 in the competitive and 0.32/100,000 in the non-competitive population). The relative risk of SCD-A is 3.1 (CI 2.1–4.7; *p* < 0.0001) for competitive compared to non-competitive athletes; 9.9 for male (CI 4.6–21.4; *p* < 0.0001) with respect to female. *Conclusions:* We provided an updated incidence rate of SCD-A in both competitive and non-competitive sport in Italy. A higher risk of SCD-A among competitive and male athletes was confirmed, thus corroborating the value of Italian pre-participation screening in this population.

## 1. Introduction

Sudden cardiac death in athletes (SCD-A) is commonly defined as a death occurring within one hour of initial acute symptoms due to cardiovascular collapse (or within 24 h in unwitnessed cases) among sport practitioners in the absence of external causal factors [1]. This definition excludes deaths related to traumas, technical errors and use of drugs (doping included). SCD-A is an extremely relevant event for public health, because it is unexpected and involves people at low mortality risk, a population considered “healthy” by definition. The incidence of SCD-A in the general population [2,3,4] and among athletes [5] is hard to define and shows remarkable variability between all available studies in the literature, ranging from 0.12 events in 100,000 persons per year in the adult general population [6] to 6.8/100,000 among UK-based adolescent soccer players [7], to 6.64/100,000 among competitive athletes between 35 and 49 years old [8]. This consequently brings broad controversies about the best approach to prevent SCD-A [9].

Italian law requires pre-participation screening for competitive sport practice nationwide since 1982, aimed to certify a physical status consistent with an organized and continuative athletic activity [10]. Since then, a few meaningful studies proofed the effectiveness of this wide-scale preventive strategy. In particular, Corrado et al. in 2006 showed a clear decrease of incidence of SCD-A since the introduction of this systematic pre-participation screening [11].

On the other hand, the “Italian model” of preventive sports medicine has not been uniformly accepted by the international scientific community, due to a number of studies displaying a very low incidence of SCD-A in the athletic population, even lower than sudden cardiac death in the general population [12], a not-negligible rate of false negatives [7,13] and false positives [14] and, last but not least, the low cost-effectiveness of this kind of screening [15,16]. Factors against the Italian screening program also include the absence of a global monitoring, taking account of costs, number of evaluated athletes and how many of them are (temporarily or permanently) disqualified from sport practice. For the sake of completeness, we have to mention a recent study from Vessella et al., which showed that in 5910, athletes who underwent the Italian pre-participation screening, a 2% of unknown diseases in apparently healthy athletes was detected with an average cost of 79€ for each evaluation [17]. Noteworthy, 18 subjects in this study were diagnosed with a cardiovascular condition at risk for SCD-A.

Above all, some concern may arise from the absence of a nationwide registry of SCD-A in Italy: from this point of view, not only data from competitive sport practice but also from the non-competitive and recreational sport should be regularly and extensively collected. A huge part of the general population practicing sport as a leisure activity, indeed, has no screening at all.

In order to fill this gap, we tried to evaluate the incidence of SCD-A nationwide using the most powerful and feasible instrument: the internet.

## 2. Materials and Methods

Since 1 January 2019 on a weekly basis, we performed a combined search on Google’s search engine of the following keywords in Italian language: “death”, “sudden”, “sport” as mandatory words (using logical AND operator), together with “match”, “race”, “training”, “certificate”, “autopsy” as non-mandatory words (using logical OR operator). At the end of the year 2019, we strengthened the results of the search adding to mandatory keywords the year of interest and the name of each of the 107 Italian provinces (e.g., “sudden death sport 2019 Agrigento”). We selected the results according to the previously provided definition of SCD-A [1], and thus, we excluded all cases of resuscitated sudden cardiac arrest. Ultimately, we performed the same search in the main foreign languages (i.e., English, Spanish, German, French) in order to ensure that any exceptional case of SCD-A which may have occurred abroad had been identified.

For every event, we collected the following data: age, date, practiced sport, level of sport participation (competitive, non-competitive), likely diagnosis and outcome of the autopsy (where available).

For this purpose, we referred to the definition of competitive sport from the 16th Bethesda conference, which defines a “competitive athlete” as an individual who participates in an organized team or in a sport that requires regular competition against others, places a high premium on excellence and achievement, and requires some form of systematic and intense training [18]. Furthermore, “non-competitive athletes” refers to individuals engaged in recreational or leisure-time sports activities, either on a regular basis or intermittently [4].

Regarding state-of-the-art sport practice in Italy, the main and most recent sources of sport-related statistical data are represented by the Annual Report developed by the Italian National Institute of Statistics (ISTAT) [19] and by the Italian National Olympic Committee (CONI) monitoring made through the “Centro Studi CONI Servizi” [20], an overview of the organized sport movement in the Italian landscape. According to ISTAT data, 35.3% of the whole Italian population aged over 3 years (20.680 million of 58.583 million people) claim to practice one or more sports in their spare time; 11.719 million of them are men, and 8.961 million are women. Regarding competitive sport practice, according to CONI data, there are 4.703 million athletes among Italian sporting bodies, equal to 8.1% of all Italians aged 3 years or older. Among them, 71.8% (3.377 million people) are men and 28.2% (1.326 million people) are women.

In summary, we can state that today in Italy, there are about 4.703 million competitive athletes and about 15.977 million non-competitive athletes (8.342 million men and 7.635 million women).

### Statistical Analysis

Quantitative variables are presented as mean ± standard deviation, categorical variables as count and percentage. The only comparisons were made on categorical variables (i.e., age, sex, level of sport participation), and chi square test was used. The software SPSS 25.0 for Mac was used for statistical analysis. A difference was considered significant when *p* ≤ 0.05.

## 3. Results

Following our Google-based research, we identified 98 cases of SCD-A on the Italian soil in the year 2019. Among them, 47 (48.0%) involved people practicing competitive sport, whereas 51 (52.0%) involved non-competitive athletes. These events concerned far more male people (92.9%), with a male-to-female ratio of about 13:1 (*p* < 0.0001). In particular, we found 46 events among male competitive athletes (97.9% of the events in the competitive population, 46.9% of the total number of events) and only 1 event among female competitive athletes (2.1% of the events in the competitive population, 1.0% of the total). Conversely, in the non-competitive population, we detected 45 male deaths (88.2% of the events in the non-competitive population, 45.9% of the total) and 6 female deaths (12% of the events in the non-competitive population, 6.1% of the total).

**Figure 1:**
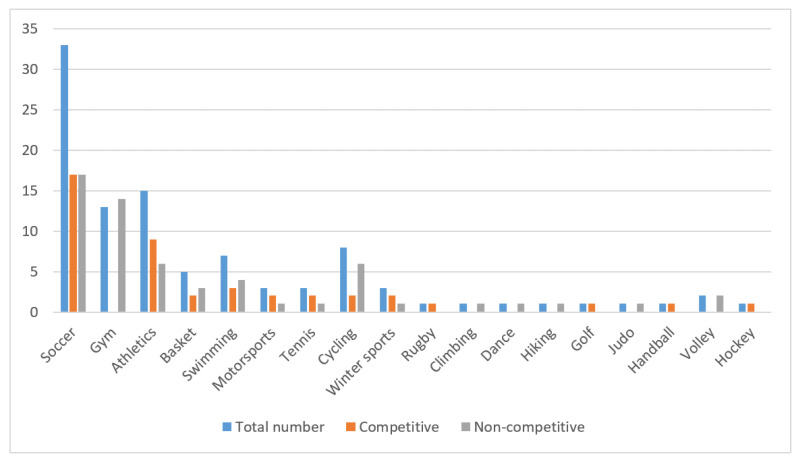
Occurrences of sudden cardiac death in athletes during 2019 in Italy, classified by sport and individual level of participation.

According to our data (Figure 1), the most frequently involved sports in SCD-A cases were soccer, with 33 occurrences (33.7%); athletics (especially running) with 15 cases (15.3%), and fitness activities in the gym, with 13 fatalities (13.3%).

Events happened in 50 cases (51%) during sport practice, in 9 cases (9.2%) at the end of the sport activity, in 14 cases (14.3%) during the night-time sleep and in 24 cases (24.5%) at rest or during the day-to-day activities. Only in one case of SCD-A could circumstances not be established.

In terms of time of the SCD-A events, the mostly involved months of the year were February (12.2%), September (10.2%) and October (17.3%); however, there were no significant differences among months.

**Figure 2:**
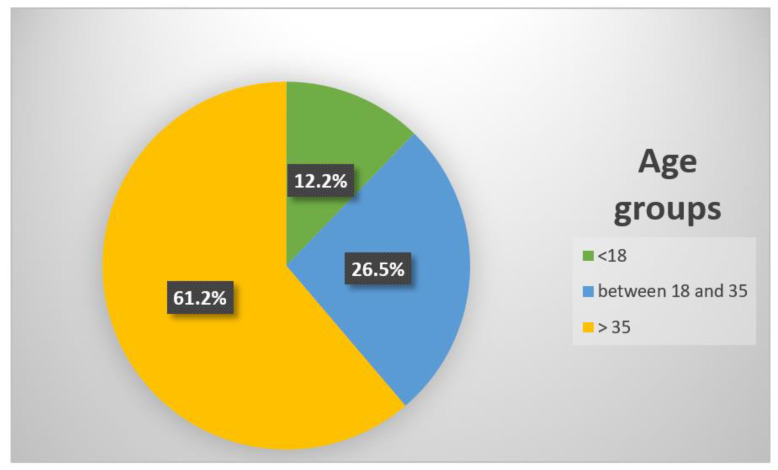
Occurrences of sudden cardiac death in athletes during 2019 in Italy, classified by age groups.

Classifying events by age groups, we found 12 episodes among under 18-year-old people (12.2%), 26 among people between 18 and 35 years old (26.5%) and 60 among people older than 35 years old (61.2%) (Figure 2). More specifically, in the competitive subgroup, there were 8 cases (17.0%) among people under 18 years old, 14 (29.8%) among people between 18 and 35 years old and 25 (53.2%) among people older than 35 years old; in the non-competitive subgroup, the number of events in the same age groups were 4 (7.8%), 12 (23.5%) and 35 (68.6%) respectively.

**Figure 3:**
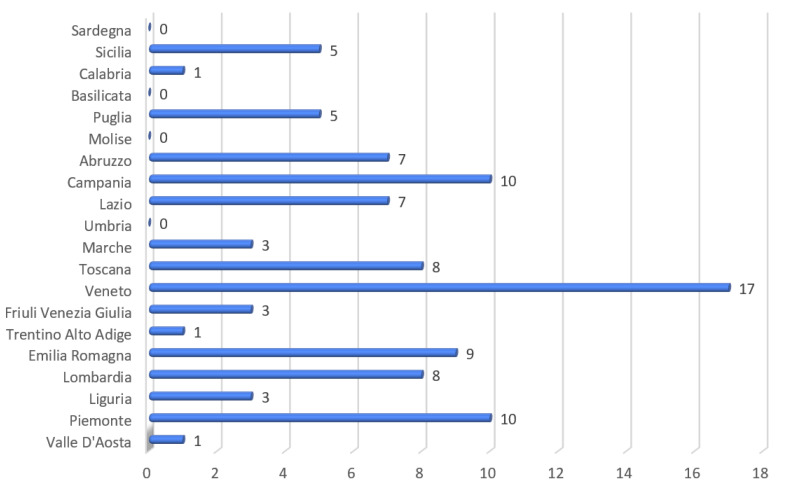
Number of cases of sudden cardiac death in athletes in each Italian Region during 2019.

Considering the country administrative subdivision in Regions, we delineated the number of events for every Italian Region in Figure 3.

Concerning etiopathogenetical definition of SCD-A cases, in 61 cases (62.2%), we achieved only a probable diagnosis of the cause of death (based on reported information about circumstance of death and medical history, but without any autopsy report); conversely, we attained a conclusive diagnosis in 37 cases (37.8%), with a cardiac origin in 33 (33.7%). The leading diagnosis was coronary artery disease (CAD) in 27 cases (73.0%), followed by acute myopericarditis in two (5.4%), one case (2.7%) of anomalous origin of a coronary artery (single coronary artery), one of arrhythmogenic cardiomyopathy (2.7%), one of pulmonary embolism (2.7%) and one of acute heart failure (2.7%). In the remaining four cases, the SCD-A had a non-cardiac origin (i.e., cerebral hemorrhage in 2, bacterial meningitis in one and acute respiratory failure in one).

The most valuable etiological subset was clearly CAD: this subgroup was composed totally (100%) of men, 26 out of 27 (96.3%) aged 35 years or older (*p* < 0.0001 in respect to athletes younger than 35), with a mean age of 51.1 years (±11.06 years). There were 10 competitive athletes in this subset (37.0%), whereas 17 were non-competitive athletes (63.0%) (*p* = 0.27).

Combining our data with ISTAT and CONI reports, the SCD-A incidence rate in the whole sport population was equal to 0.47 in 100,000 persons per year, 0.78 among male and 0.08 among female athletes (*p* < 0.0001). In the competitive sport population, it was 1.00/100,000 persons per year (1.36 in male and 0.08 in female, *p* < 0.0001), whereas in the non-competitive sport population, it was 0.32 (0.54 in men and 0.08 in women, *p* < 0.0001).

The relative risk (RR) of SCD-A for competitive athletes was 3.1 (CI 2.1–4.7; *p* < 0.0001) compared to non-competitive athletes; it was 2.5 in males (CI 1.7–3.8; *p* < 0.0001) and 1.0 in females (CI 0.1–8.0, *p* = 0.96).

Compared to female athletes, male athletes showed a RR of SCD-A of 9.9 (CI 4.6–21.4; *p* < 0.0001) and in the whole sport population of 18.0 (CI 2.5–131.0; *p* = 0.0042) in the competitive population and of 6.9 (CI 2.9–16.1; *p* < 0.0001) among non-competitive practitioners.

## 4. Discussion

A proper definition of SCD-A cases is a crucial factor to determine the real incidence of these events and subsequently to evaluate the best pre-participation screening strategies and their efficacy through time. There is still a lack of consensus in this regard: some authors consider the whole athletic population who suffer from sudden cardiac death, [7,8,21,22] whereas others account only for deaths that happened during sport practice or within 1 h of cessation of sport activity [2,6,12].

In this study, we adopted the first definition as the most all-encompassing in order to take into consideration every possible sport-related cause of sudden cardiac death, which may not exclusively manifest in direct correspondence with sport activity, but in which sport practice may play a triggering role. In other words, our target has not been to identify only the events directly caused by sport, but every occurrence in an athletic population, in which sport activity might be involved as a precipitating factor. To be thorough, using the more restrictive definition of SCD-A, we have to report that the eligible events would have been equal to 58 (23 competitive and 35 non-competitive), with a SCD-A incidence rate of 0.28/100,000 in the whole population, 0.49/100,000 among competitive and 0.22/100,000 among non-competitive practitioners.

Literature is unfortunately rich with different epidemiological studies on SCD-A, with regard to aforementioned definitions used, considering population (athletes of different age [8,23], high school [21,24] and college [25] athletes, military personnel [26], competitive and non-competitive practitioners [2,27], general sport population [2,6]) or study design (cross-sectional survey [24], prospective [2,6,7], retrospective [23,24,25,26,27]). A recent review [28], summarizing the results of 24 observational studies between 2003 and 2016, outlined an incidence rate of SCD-A among the general sporting population within a range of 0.12 to 0.46 in 100,000 person per year. This fits together with our findings, which show an incidence rate of 0.47 in 100,000 persons per year in the same population. Another review in 2014 reported a little higher annual incidence of SCD-A in competitive athletes ranging from 1/40,000 to 1/80,000 (i.e., 1.7/100,000) [29].

As for Italy, epidemiological data on SCD-A primarily ensued from a Corrado et al. study [22], which prospectively examined SCD-A events in a youth population (between 12 and 35 years of age) in the Veneto region of Italy, demonstrating an incidence rate equal to 1.0 in 100,000 persons per year, including 2.3/100,000 among competitive athletes (similar to what reported by Harmon et al. [29]) and 0.9 among non-athletic population. These data, albeit extremely accurate, are clearly limited to one Italian region and do not take into account the large number of master athletes (over 35 years old) practicing competitive sport today; moreover, they analyze only people submitted to Italian mandatory pre-participation screening (i.e., competitive athletes), excluding leisure-time practitioners. Our study tries to fill this gap, taking into consideration also master and leisure-time athletes. Although designed with a different aim (to determine the impact of early defibrillation in all cases of sudden cardiac arrest occurring in sport facilities), a recent work by Zorzi et al. [30] had already used our internet-based search methodology profitably in this field, identifying 123 cases of sudden cardiac arrests in Italian sport facilities during 2015.

Analyzing the young athletic population (aged 12 to 35 years old), estimated by ISTAT in about 14 million 874 thousand people, we found 37 occurrences in our series, with an incidence rate of SCD-A of 0.25 in 100,000 persons per year: these figures are 4 times lower than incidence rate determined by Corrado et al., but they match with data from Marjion et al. (0.22/100,000) [2] and Risgaard et al. (0.43/100,000) [8]. In any case, our series corroborates the evidence of a substantially increased risk of SCD-A in competitive athletes compared to non-competitive people, with an RR of 3.1 (CI 2.1–4.7; *p* < 0.0001). This risk is very close to the risk demonstrated by Corrado et al. (2.5, CI 1.8–3.4) between competitive and non-athletic population, even if this comparison is somehow different with respect to our data. Whilst we compared competitive and non-competitive athletes, Corrado et al. compared competitive athletes and the non-athletic population, where both sedentary subjects and non-competitive athletes were included, being non-competitive athletes at higher risk of SCD-A. Finally, we cannot exclude that in our series, some of the non-competitive athletes affected by SCD-A were previously disqualified by competitive practice, according to Italian pre-participation rules. All these data do confirm the usefulness of pre-participation screening for competitive sport.

The most obvious dissimilarity between our series and other known data regards the difference of incidence rates between genders, specifically among competitive athletes: we found an annual incidence rate in the competitive male population of 1.36/100,000 persons and 0.08 in the competitive female population, whereas Corrado et al. reported an incidence rate of 2.6 and 1.1/100,000, respectively. However, Corrado’s population comprised young people (12–35 years), whilst our data included master athletes, with a considerable number of SCD-A to be linked to CAD, especially among males [31,32]. This discordance between genders has been pointed out in other studies, but there is a great difference in magnitude: Marjion et al. [2] outlined an incidence of 0.92 in male and 0.04 in female among the general population, and Bohm et al. reported an incidence among the general population over 18 years old of 0.16 in 100,000 male and 0.006 in 100,000 female [6]; on the contrary, a slighter difference was found by Harmon et al. among young competitive athletes (2.63 in 100,000 male vs. 0.82 in 100,000 female per year) [25] and by Eckart et al. among active military personnel over 18 years of age (6.7 in 100,000 male and 1.4 in 100,000 in female) [26]. This seems to deserve further consideration.

Data from Italian National Olympic Committee (CONI) monitoring, through the total number of competitive athletes engaged in every sport, allowed us also to estimate the incidence of SCD-A per every competitive sport: from this perspective, we found the highest incidence of SCD-A in hockey (18.2/100,000), motorsports (9.5/100,000), athletics (3.3/100,000) and handball (3.0/100,000), as shown in Table 1.

Lastly, a well-known but always relevant aspect outlined concerns the wide difference in CAD incidence in people younger and older than 35 years old, which confirms a strong link between age and atherosclerosis-related cardiovascular diseases [31,32].

The availability of a nationwide registry in Italy of all pre-participation examination data would allow for more accurate information about competitive athletes, in terms of age groups, practiced sports, identified diseases and outcome of the examination (sport practice allowed or forbidden).

## 5. Study Limitations

An internet-based epidemiological search depends closely on completeness and authenticity of online information: this implies the possibility of errors in data collection wherever they were incorrectly reported by media.

In this respect, it is also possible that some cases of SCD-A that occurred in Italy did not get any online news coverage and, therefore, did not get included in our work. For this reason, we purposely excluded all cases of resuscitated sudden cardiac arrest, because in our opinion they are probably more prone to be under-reported by online media. Although we consider this eventuality negligible, it cannot also be excluded that some cases of SCD-A have not been detected due to discrepancy between our search keywords and keywords used by newspapers. All of these factors could lead to an underestimation of the real total number of SCD-A events in Italy, thus eventually decreasing the epidemiological value of our data. On the other hand, the several case and series reports of SCD-A published, even if showing data in a more complete and scientific fashion, miss the real number of cases even more so than a web-based approach. Some cases not fulfilling inclusion criteria or a complete study, in fact, are not reported.

Lastly, we assume irrelevant the use of the online research tool in order to identify the cause of SCD-A: in our series, a definite diagnosis was determined only in 37% of cases, reasonably due to privacy protection in compliance with current regulations. Methods that are more effective should be hereafter used in order to obtain these valuable data, possibly nationwide.

## 6. Conclusions

Our study allows us to identify a remarkable number of SCD-A events in Italy, establishing a present-day baseline incidence rate of SCD-A in both competitive and non-competitive sport practitioners.

Our series confirms available incidence rates of SCD-A in the general sport population and the higher risk of SCD-A among competitive athletes, thus corroborating the value of Italian pre-participation screening in this population.

A notably hot topic regards the huge difference in incidence we found between the two genders, especially evident among competitive athletes: this aspect seems to deserve further consideration.

Another key point that deserves more attention in the future is the adoption of an unambiguous definition of SCD-A, which should take into account, in our opinion, all sudden cardiac events in sporting people.

Further studies based on alternative approaches are certainly required to validate our findings. In any case, we consider fundamental in the near future a wide-range collection and systematic analysis of this sort of epidemiological data, taking into account both competitive and non-competitive athletes, for evaluating properly the actual impact of a pre-participation screening on the incidence rate of SCD-A. The most important target is, in our opinion, the creation of a “nationwide registry of sport-related sudden cardiac death”, which records age, practiced sport and level of participation (competitive or non-competitive), the circumstances of the event and (wherever possible) a definite cause of death.

## Figures and Tables

**Table 1:** Incidence of Sudden Cardiac Death in athletes divided per competitive sport (data from Italian National Olympic Committee, CONI).

Sport	Competitive Athletes	SCD-A in Competitive Athletes	Incidence of SCD-A Per Sport (Per 100,000 Athletes/Year)
Soccer	1,056,824	17	1.6
Tennis	372,964	2	0.5
Basketball	317,321	2	0.6
Athletics	270,602	9	3.3
Swimming	163,307	3	1.8
Golf	90,167	1	1.1
Rugby	82,432	1	1.2
Cycling	75,543	2	2.6
Winter sports	73,541	2	2.7
Handball	33,021	1	3.3
Motorsports	20,958	2	9.5
Hockey	5509	1	18.2

## Data Availability

The data presented in this study are available on request from the corresponding author. The data are not publicly available because they involve sensitive informations.
